# Global research status and emerging frontiers of Shoutai Wan in the prevention and treatment of spontaneous abortion: a bibliometric analysis (1982–2026)

**DOI:** 10.3389/fmed.2026.1850663

**Published:** 2026-10-13

**Authors:** Meng Tan, Si Chen, Ting Wang, Han-qing Zhao, Mei-li Chen, Song-ping Luo, Hai-wang Wu

**Affiliations:** 1 The First Clinical College, Guangzhou University of Chinese Medicine, Guangzhou, China; 2 The First Affiliated Hospital of Guangzhou University of Chinese Medicine, Guangzhou, China; 3 Guangdong Clinical Research Academy of Chinese Medicine, Guangzhou, China; 4 University of Hebei, Baoding, China

**Keywords:** bibliometric analysis, Shoutai Wan, spontaneous abortion, traditional Chinese medicine, visualization

## Abstract

**Objective:**

This study representing the most comprehensive bibliometric analysis to date on Shoutai Wan (STW) in the field of spontaneous abortion aimed to systematically analyze the research hotspots and frontiers of STW in spontaneous abortion (SA) prevention and treatment using bibliometric methods and provide references for future basic mechanism research and clinical applications.

**Methods:**

Relevant literature published from inception to January 2026 was retrieved from the Web of Science, PubMed, CNKI, VIP, and Wanfang databases. CiteSpace 6.4R1, VOSviewer 1.6.20, and Origin 2025 were used to analyze journal literature on STW in the prevention and treatment over the past 40 years, followed by a qualitative analysis and evaluation.

**Results:**

This study involved a comprehensive analysis of 255 articles. STW publications stagnated at a low level from 1982 to 2007, exhibiting overall fluctuating growth after 2008, with a notable surge in mechanistic investigations over the past 5 years. Luo SP, Gao J, Lei L, and Du HL were key figures. Guangzhou University of Chinese Medicine, Hunan University of Chinese Medicine, and Hebei University of Chinese Medicine served as the three primary hubs for “STW-mechanism research.” China was the leading country. Threatened and recurrent abortion, the most common subtypes of SA, exhibited the most significant association with STW. Keywords with high frequency and high betweenness centrality included kidney deficiency syndrome, progesterone, clinical efficacy, inflammatory factors, IL-17, HSP27 and aerobic glycolysis. Keyword clustering analysis identified three main research foci: Clinical efficacy assessment, investigation of critical mechanisms such as immune modulation, oxidative stress, and apoptosis, and the impact of STW on the maternal-fetal interface.

**Conclusion:**

STW research is transitioning from “clinical empirical application” to balancing evidence-based practice and mechanism clarification. However, challenges persist, including insufficient clinical evidence, fragmented mechanism studies, and a disparity between traditional Chinese medicine (TCM) macro theory and micro mechanism. Future research should prioritize high-quality multicenter clinical trials, developing a TCM-mechanism-target model, and using multi-omics for maternal-fetal biology mapping.

## 1 Introduction

In China, spontaneous abortion (SA) is defined as the loss of an intrauterine pregnancy before 28 weeks of gestation, with the fetus weighing <1,000 g at birth (1). However, the Royal College of Obstetricians and Gynaecologists (RCOG) and the European Society of Human Reproduction and Embryology (ESHRE) define SA as pregnancy loss occurring before 24 weeks of gestation (2, 3). There is a lack of consensus on the inclusion of biochemical pregnancy in SA management, and this category is excluded from the current study's scope. Pregnancy loss is a significant global challenge in female reproductive health (2), with SA being a primary contributing factor. The prevalence of SA among women of reproductive age is estimated to range from 10% to 15%, with a propensity for recurrence (3).

Shoutai Wan (STW), derived from Zhang Xichun's Qing Dynasty medical text, combines *Cuscuta chinensis* (dodder seed), *Dipsacus asperoides* (teasel root), *Taxillus chinensis* (mistletoe), and *Colla Corii Asini* (donkey-hide gelatin). It is renowned for kidney-tonifying and miscarriage prevention, earning the title “a sacred formula for miscarriage prevention.” (4) STW's robust theoretical foundation and extensive clinical application render it a pivotal option for SA in TCM. Despite an increasing number of mechanistic and clinical studies, few have systematically analyzed STW's role in SA prevention and treatment using visualization tools, leaving research hotspots and frontiers unclear.

To the best of our knowledge, few studies have systematically mapped the clinical and mechanistic literature on STW in spontaneous abortion across multiple databases and analytical dimensions. Among the two published bibliometric studies on STW, one (5) was limited to literature sourced from four Chinese domestic databases, specifically CNKI, Wanfang Data, VIP, and the Chinese Biomedical Literature Database (CBM), with a search cutoff in November 2024. This study encompassed 794 articles, none of which were published in English. Similarly, the other (6) focused solely on the CNKI database, covering publications from January 2000 to August 2023, and included 599 articles, also excluding English-language literature. In contrast, the present study retrieved records from five databases, both Chinese and English—Web of Science, PubMed, CNKI, VIP, and Wanfang Data—spanning a period of 44 years from 1982 to 2026. This renders our study the most current and data-inclusive bibliometric analysis on STW, thoroughly mapping its progression from empirical clinical application to detailed mechanistic exploration. Unlike previous bibliometric analyses that primarily identified general research hotspots (5, 6), we employed two well-established bibliometric visualization techniques, CiteSpace and VOSviewer, to systematically collate and critically assess studies focusing on STW for SA prevention and clinical management, with the literature retrieval cutoff set to March 2026. We conducted a comprehensive analysis of the historical progression of research related to STW, noting a transition from an initial focus on clinical efficacy to more recent investigations into the pharmacological mechanisms of modified STW formulations. Key molecular targets of STW were precisely identified, including the PI3 K/protein kinase B (PKB), MAPK, FOXO3-PR, STAT3, and nuclear factor-κB (NF-κB) pathways. Utilizing clustering analysis, we categorized the underlying mechanisms into three functional modules: immunological regulation, inhibition of cellular apoptosis, and enhancement of microcirculation at the maternal-fetal interface (MFI).

## 2 Materials and methods

### 2.1 Data sources and retrieval strategies

Data were obtained from Web of Science, PubMed, CNKI, VIP Database, and Wanfang Database. A targeted and comprehensive search strategy focusing on “spontaneous abortion” and “Shoutai Wan” was formulated, with complete details provided in Supplementary Table S1. The final search was conducted on January 30, 2026, to maintain the stability of our literature pool and mitigate biases arising from database updates.

### 2.2 Inclusion and exclusion criteria

The inclusion criteria included experimental and clinical studies related to SA treatment with STW; the prescriptions used in studies employed STW as the core formula, incorporating no more than three supplementary medicinal herbs for modification.

The exclusion criteria were as follows: (1) Non-original research, including literature studies, systematic reviews, and meta-analyses; (2) dissertations, conference proceedings, advertisements, and newspapers; (3) duplicate publications or retracted articles; (4) literature with unavailable key information, including authors, publication years, and keywords.

### 2.3 Data management

Data cleaning aims to standardize synonymous keywords and remove irrelevant ones. We exported the retrieved articles in plain text format, including complete records and references.

#### 2.3.1 Deduplication protocol

After exporting records, duplicates were removed using EndNote X9 (primary) and manual verification (secondary). The matching criteria were: (a) identical DOI; (b) identical title + first author; (c) for Chinese databases (CNKI, VIP, Wanfang), title normalized via removal of punctuation and case conversion, followed by fuzzy matching with a similarity threshold *≥ 95%*.

#### 2.3.2 Author-name standardization

Variants of the same author name (e.g., “Luo SP”, “Luo Songping”, “Luo S”) were manually mapped using institutional affiliation and co-author networks as disambiguation keys. This mapping was cross-validated against the Web of Science Author ID where available.

#### 2.3.3 Institutional-name harmonization

Institutional names were normalized using a predefined thesaurus (e.g., “Guangzhou Univ Chin Med” → “Guangzhou University of Chinese Medicine”; “1st Affil Hosp” → “First Affiliated Hospital”). All affiliated hospitals of a given university were aggregated under the parent institution for network analysis.

#### 2.3.4 Cross-database metadata integration

For Chinese-language articles indexed in both WoS/PubMed and domestic databases, metadata (keywords, abstracts) were merged by matching on title. English keywords from WoS/PubMed were prioritized; Chinese keywords from CNKI/VIP/Wanfang were translated and merged after synonym resolution (e.g., “recurrent abortion” and “recurrent miscarriage” were retained as separate but cross-referenced terms). Redundant words resulting from translation duplication were systematically removed.

### 2.4 Software parameter disclosure

The plain text format was subsequently imported into visualization analysis software tools, specifically CiteSpace 6.4 R1 and VOSviewer 1.6.20. For the analysis conducted using CiteSpace 6.4 R1, the time span was set from 2008 to 2026 with a time slice length of one year. The g-index (*k* *=* *25*) was employed to filter the literature, and the Pathfinder algorithm was utilized to prune the network. The resulting visualized network comprised 216 nodes and 328 connections, yielding a network density of *0.0141*. The network's modularity was calculated to be *Q* *=* *0.8556*, with a weighted average silhouette coefficient of *S* *=* *0.9748*. In the analysis using VOSviewer 1.6.20, association strength normalization was applied, with the minimum frequency threshold for keyword co-occurrence set at four. The default VOS clustering algorithm was utilized for clustering.

CiteSpace focuses on frontier identification and burst detection, while VOSviewer is good at co-occurrence clustering and visualization. The combined use of the two tools conforms to the general specifications of bibliometrics and can ensure the comprehensiveness and reliability of the analysis. In this context, we analyzed and evaluated the visualized results and conducted a qualitative analysis with reference to established methodological recommendations.

## 3 Results and analytical discussion

### 3.1 Analysis of the number of publications

A total of 2,220 Chinese articles and 69 English articles were retrieved utilizing the retrieval strategy. After the exclusion of 2,034 articles, 255 articles that met the inclusion and exclusion criteria were obtained. After the exclusion of 2,034 articles, 255 articles that met the inclusion and exclusion criteria were obtained. Among the included literature in this study, basic research (such as *in vivo* and *in vitro* experiments, signal pathway exploration, etc.) accounts for 47%, and clinical research accounts for 53%. Figure 1 illustrates the comprehensive process of article selection.

**Figure 1:**
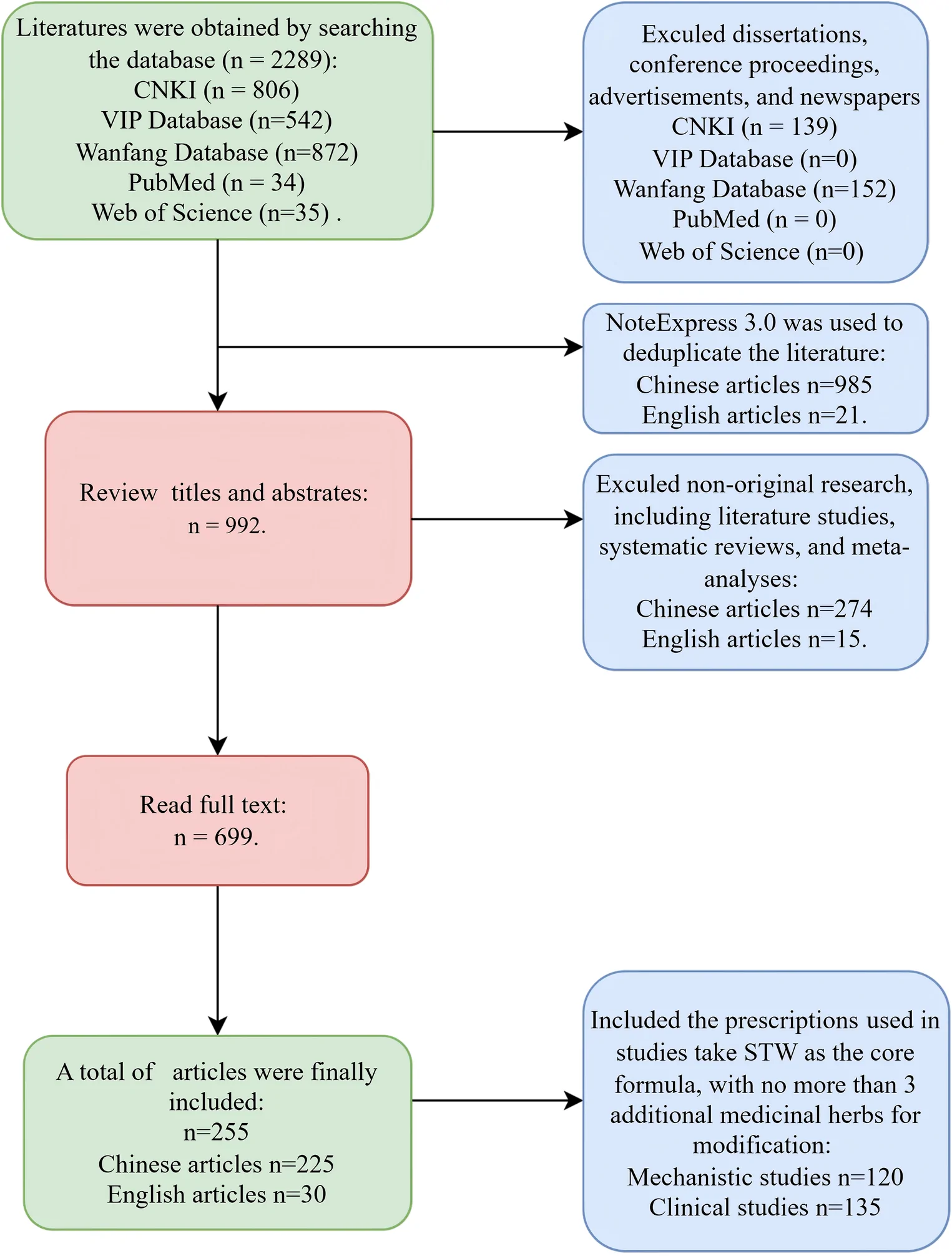
Flowchart of literature screening.

Data were input into Origin 2025 to illustrate the temporal trends and structural changes in STW-related publications from 1982 to 2026, as displayed in Figure 2. Publication activity comprised two phases. Between 1982 and 2007, annual publications were consistently modest, indicating a phase of initial research and progressive accumulation. After 2008, the volume increased significantly, with rapid growth commencing in 2010. A peak occurred in 2024, with 21 publications constituting 8.2% of the total. Linear regression analysis (excluding 2026 data) yielded an *R*^2^ of *0.7808*, indicating a significant linear growth trend and sustained research interest.

**Figure 2:**
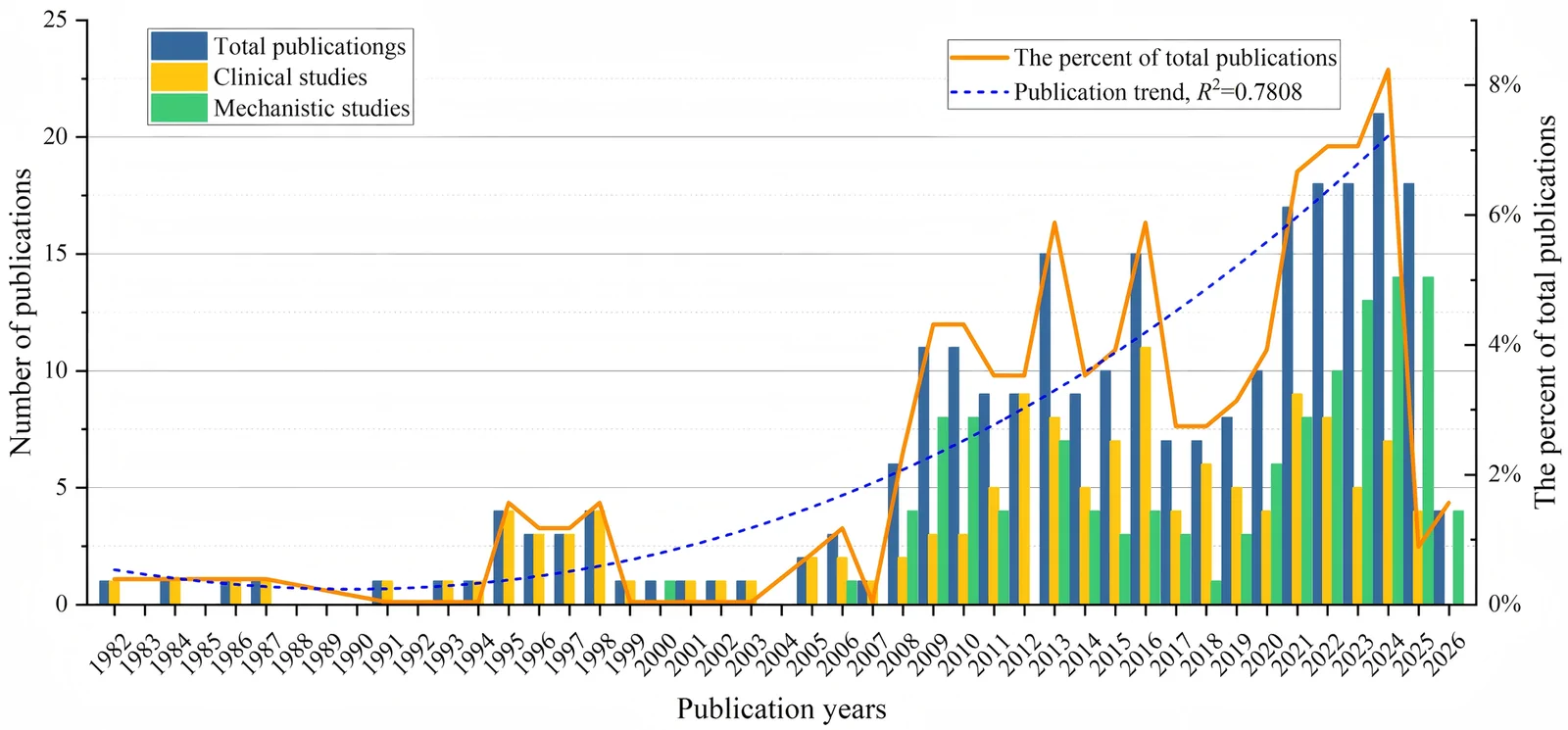
Trend chart of the annual inclusion from 1992 to 2026.

Regarding research structure, clinical studies have consistently dominated the publication field, reflecting the overall growth in research. This highlights a persistent emphasis on evaluating STW's therapeutic application and efficacy. Before 2008, mechanistic studies were rare, but they surged post-2010. Since 2020, mechanistic research has grown rapidly to nearly match clinical studies, indicative of a gradual shift in the literature from clinical observations to experimental investigations of potential mechanisms. Certainly, it must be emphasized that the core significance of this analysis lies in uncovering the evolving trajectory of research priorities across time. This temporal pattern principally reflects shifting thematic emphases within the scholarly literature, rather than serving as definitive evidence that the underlying mechanisms have been conclusively elucidated.

### 3.2 Co-authorship, co-institution, and co-country analysis

Figure 3A clearly depicts the author collaboration network, illustrating a distinct core-periphery structure. It is constructed on several long-lasting, stable teams, each focused on core scholars. Over time, a nascent research network has emerged, with Luo SP, Gao J, Lei L, and Du HL as key figures in STW studies.

**Figure 3:**
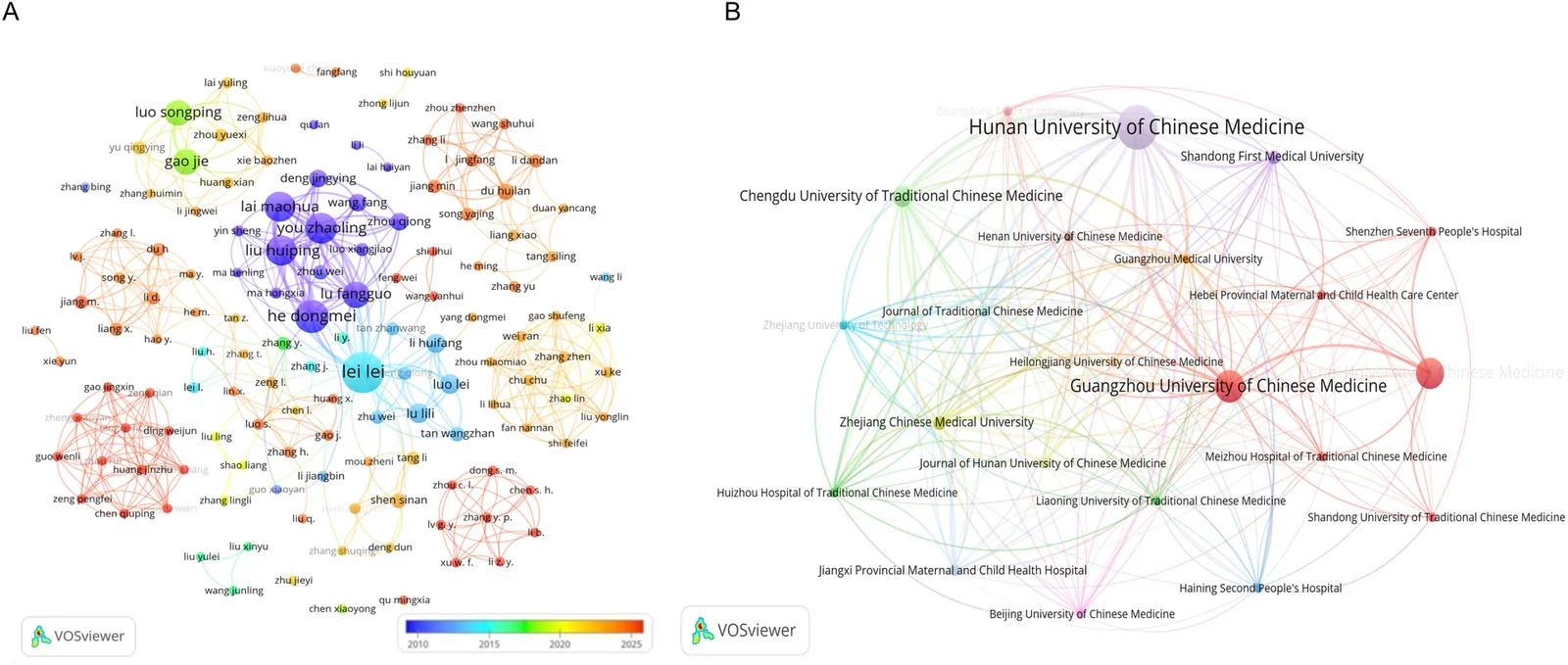
**(A)** Network map of the co-authorship. **(B)** Network map of the co-institution.

As illustrated in Figure 3B and Table 1 and Supplementary S2, S3, Guangzhou University of Chinese Medicine, Hunan University of Chinese Medicine, and Hebei University of Chinese Medicine serve as the three primary hubs for “STW-mechanism research.” Collaboration is predominantly regional, marked by robust relationships among universities and their affiliated hospitals, although cross-regional cooperation remains limited. In summarizing research teams, all affiliated institutions for each university were included in the total frequency for that university. Research activities are predominantly conducted at numerous Chinese medicine universities and their affiliated hospitals. Core collaboration networks are predominantly located in regions including Hunan, Hebei, and Guangdong, with most studies conducted in a single-center format. The regionality of clinical samples significantly constrains the scope of research, resulting in a lack of large-scale, multicenter collaborative studies and consequently leading to insufficient clinical evidence-based rigor.

**Table 1:** Members, collaborating institutions, and research directions of the top 3 author teams by publication count.

ID	Team	Coremembers	Main research directions
1	Luo SP team of Guangzhou University of Traditional Chinese Medicine	Luo SP, Gao J, Yu QY and so on	The team has pioneered research on simplified Modified STW (STW without Ejiao). They've clarified its roles in immunomodulation (7) and angiogenesis (8, 9), advancing formula optimization and mechanistic study of TCM compounds (10, 11).
2	Lei Lei team of Hunan University of Traditional Chinese Medicine	Lei L, Wang YH, Feng W, etc.	The team's research covers multiple directions, including oxidative stress, autophagy, metabolomics (12, 13) and so on, establishing a multi-target action system of STW.
3	Du Huilan team of Hebei University of Traditional Chinese Medicine	Du HL, Jiang M, Lv JF and so on	The team has conducted studies on decidualization and glycolysis (14, 15). They've clarified STW's effect on endometrial receptivity and enriched the TCM concept of “kidney governing reproduction”.

The geographic distribution map (Figure 4) illustrated China's absolute dominance in this field, with research activities predominantly concentrated domestically. International collaborations were limited, and the global impact of STW research remained relatively weak.

**Figure 4:**
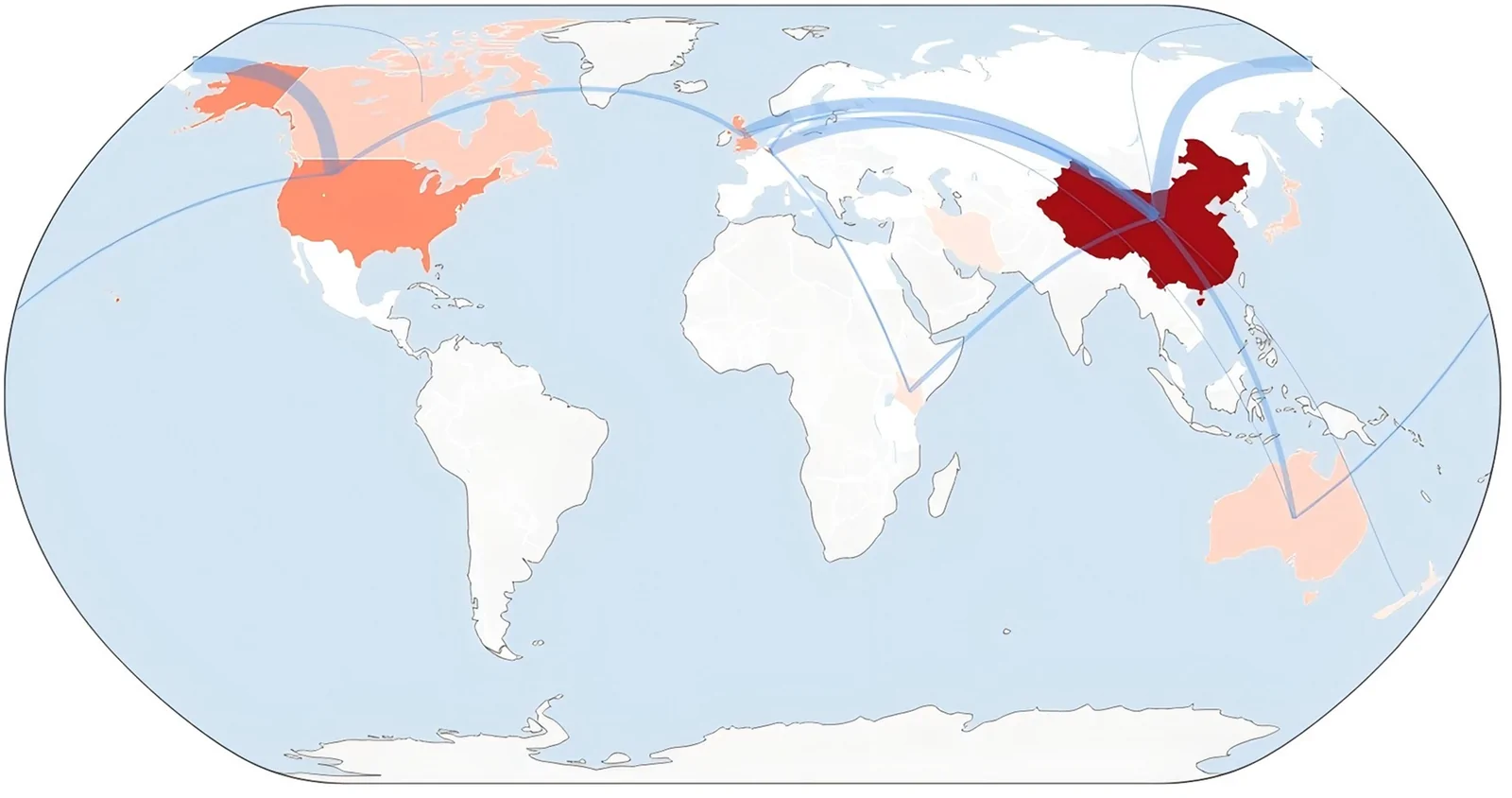
Network map of the co-country.

### 3.3 Keyword analysis

#### 3.3.1 Keyword density and keyword clustering analysis

The keyword co-occurrence map (Figure 5 and Supplementary Table S4) depicts that threatened and recurrent abortion, the most common subtypes of SA, exhibit the highest association with STW, designating them as key disease domains with broad applicability and extensive research focus. Further analysis indicates that kidney deficiency syndrome, progesterone (P), and clinical efficacy are prevalent in clinical evaluations. Mechanisms investigations focus on inflammatory factors, including interleukin (IL)-17, heat shock protein 27, aerobic glycolysis, and annexin A2, with the decidua and trophoblast cells as main action sites.

**Figure 5:**
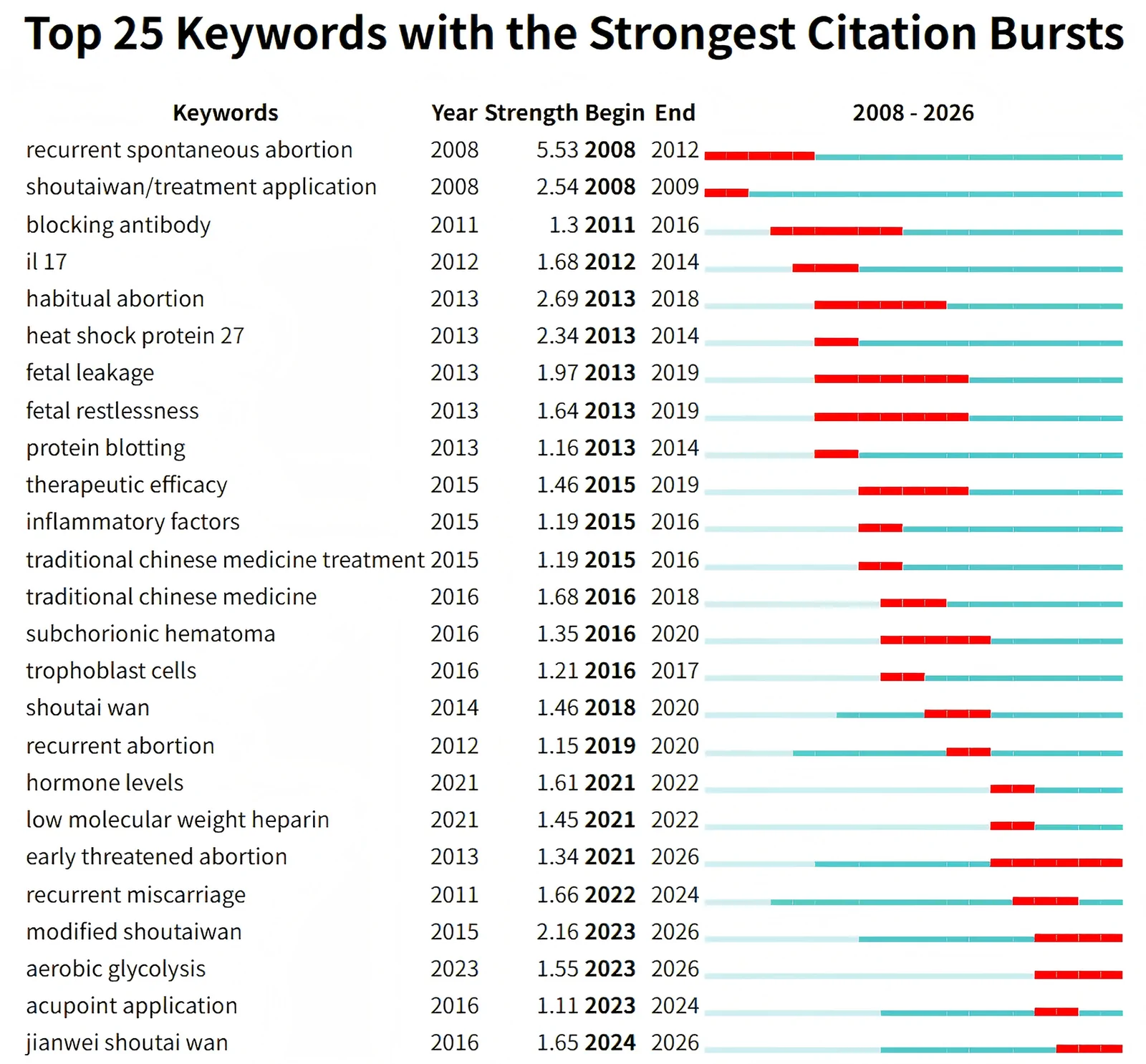
Keyword co-occurrence map of STW research (keywords with occurrence frequency >4).

Subsequent cluster analysis identified 9 unique clusters encompassing topics, as illustrated in Figure 6, including #0 recurrent abortion, #1 spontaneous abortion, #2 shoutai wan, #3 Traditional Chinese Medicine, #4 maternal-fetal interface, #5 acupoint application, #6 decidua, #7 hormone levels, #8 oxidative stress.

**Figure 6:**
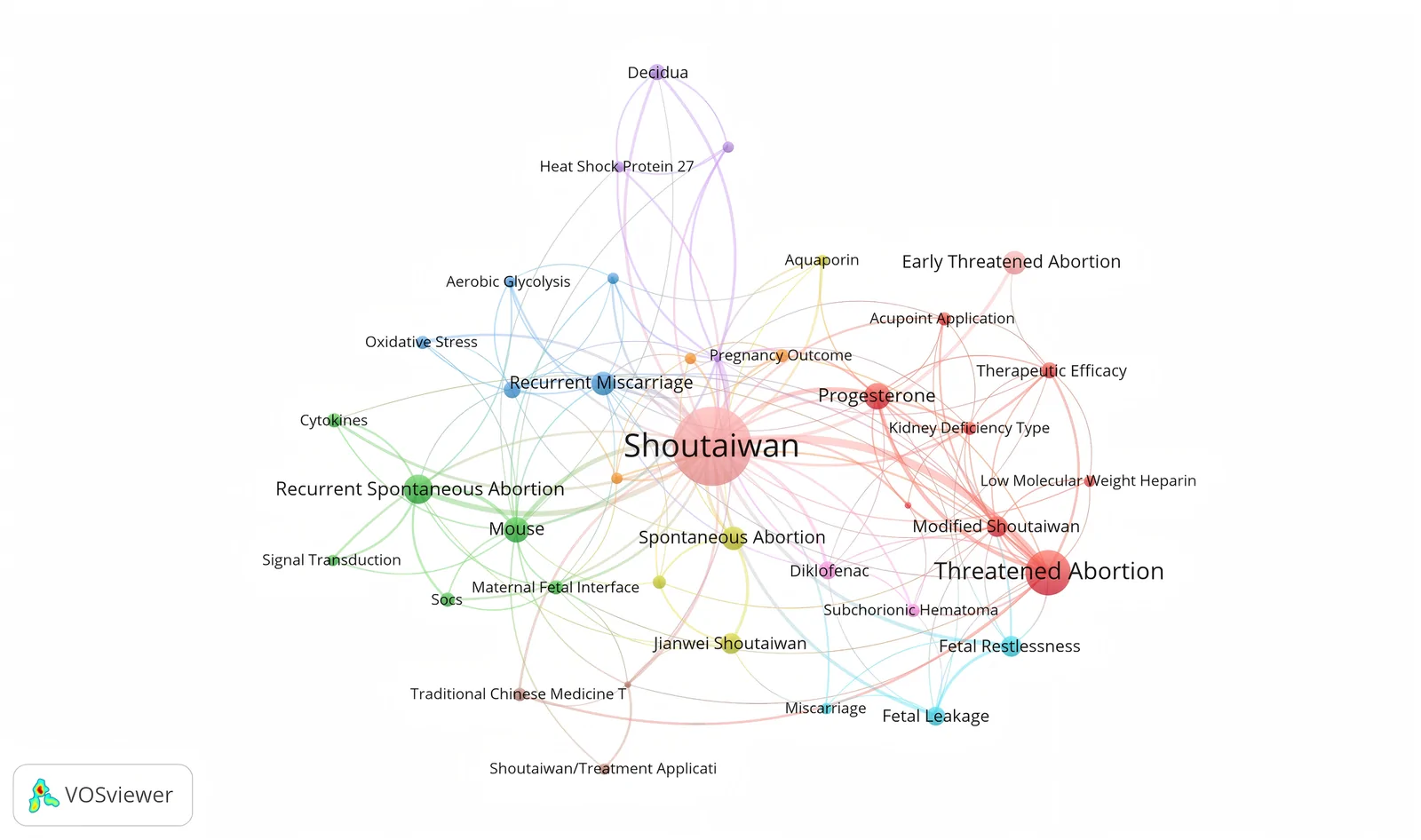
Clustering map of keywords in STW research.

#### 3.3.2 Dynamic evolution of keywords, keyword timeline, and keywords with the strongest citation bursts analysis

The time-overlay visualization network generated by VOSviewer depicts the evolving trends in research within this field. Figure 7A depicts a continuous evolutionary trajectory: initial studies (blue nodes) predominantly focused on clinical applications, whereas more recent investigations (red nodes) have progressively transitioned toward mechanistic analyses and omics technologies.

**Figure 7:**
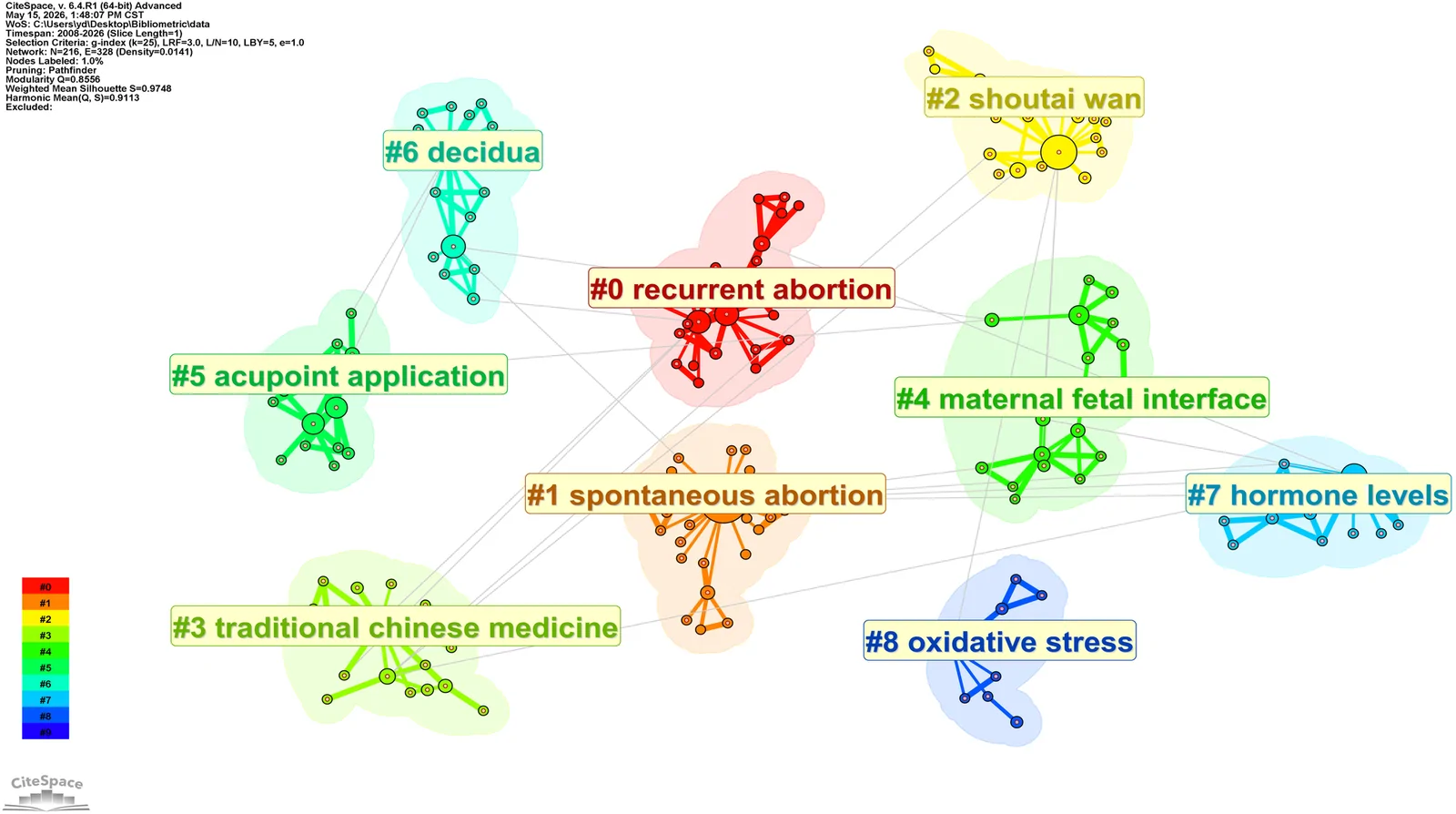
**(A)** Evolution of keywords in STW. **(B)** Time series chart of keyword clustering in STW research from 2008 to 2026.

Figure 7B and Supplementary Table S5 illustrate a timeline cluster analysis derived from literature published between 2008 and 2026. The findings reveal three distinct phases: Early phase (2008–2013): Research concentrated on therapeutic applications, focusing on STW for the treatment of threatened abortion and recurrent abortion as the core focus. Middle phase (2014–2020): This period experienced increased attention to the maternal-fetal interface, immunological modulation, and molecular mechanisms. Recent phase (2021–2026): Cutting-edge hotspots have shifted to “aerobic glycolysis” and “modified shoutai wan” (“jianwei shoutai wan”), indicating novel directions in formula modification, metabolic regulation, and precision therapy.

Figure 8 indicates the Top 25 Keywords with the Strongest Citation Bursts, depicting the rise and decline of research hotspots from 2008 to 2026, consistent with the findings in Figure 7B. Early (2008–2013), “recurrent spontaneous abortion” (burst strength, 5.53) was the dominant hotspot, and “Shoutai Wan/treatment application” focused on STW's clinical use. Middle (2013–2020), “blocking antibody,” “inflammatory factors,” and “oxidative stress” indicated a transition to immune and mechanistic studies, whereas “therapeutic efficacy” stressed clinical outcome evaluation. Recent (2021–2026) hotspots such as “modified shoutai wan” and “aerobic glycolysis” are current frontiers, guiding future research.

**Figure 8:**
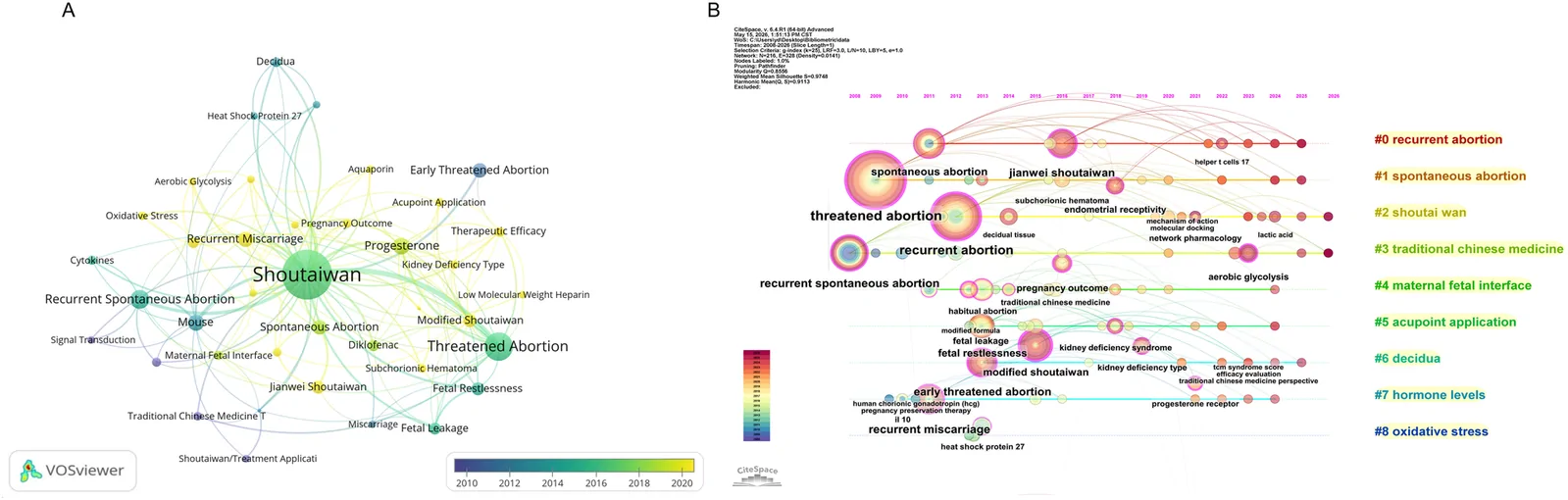
Top 25 Keywords with the Strongest Citation Bursts from 2008 to 2026.

## 4 Commentary on the research status of STW

As previously demonstrated, studies on STW concerning SA prevention and management focused on three primary domains: assessing its efficacy in treating associated conditions, investigating essential mechanisms such as immune modulation and apoptosis, and examining the effects of STW on maternal and fetal tissues and cells. We performed qualitative analysis utilizing bibliometric data to highlight the recent advancement in this domain.

### 4.1 Clinical research

STW is frequently utilized to treat various pregnancy disorders associated with kidney deficiency and is preferred by clinicians for SA prevention and treatment in the context of TCM (16). Numerous clinical trials have confirmed its safety and efficacy (17–21).

#### 4.1.1 Combined therapy with STW and western medicine

Recently, the combination of TCM with Western medicine has demonstrated significant therapeutic efficacy in clinical settings. Shi et al. (17) discovered that the combination of STW alongside dydrogesterone in treating unexplained recurrent spontaneous abortion (URSA) improved pregnancy success rates and more effectively decreased TCM syndrome scores compared to dydrogesterone monotherapy (with comprehensive drug composition and relevant conventional medicine information provided in Supplementary Table S6). Besides, by examining the discrepancies in scores on the Self-Rating Anxiety Scale and Self-Rating Depression Scale, they verified that this combination therapy might improve the mental health of patients with RSA. Yao et al. (18) conducted a randomized controlled clinical trial involving 80 patients diagnosed with threatened abortion. The control group, comprising 40 patients, received intramuscular progesterone injections, whereas the observation group, also comprising 40 patients, received STW alongside the control treatment. The findings demonstrated that the TCM syndrome score in the observation group was significantly higher than that in the control group (*P* *<* *0.05*). Furthermore, the serum levels of sex hormones, including P, estradiol (E2), and human chorionic gonadotropin (HCG), and ultrasound parameters, including hematoma area, peak systolic velocity, and end-diastolic velocity, demonstrated significant improvement in the observation group compared to the control group (*P* *<* *0.05*). These findings substantiate the conclusion that combining STW with conventional Western medical treatment yields synergistic effects, enhances the intrauterine conditions, optimizes blood flow in the uterine artery, and thereby offers a more effective therapeutic strategy for managing threatened abortion.

Additionally, numerous studies have effectively combined clinical observational research with mechanistic analyses. In a particular study (19), researchers recruited 60 women experiencing normal pregnancies and 60 patients diagnosed with URSA. The URSA cohort was stratified into two subgroups: an experimental group that received the dydrogesterone and STW combination and a control group that received only dydrogesterone. Blood samples from fasting participants were collected from the veins before and after the treatment to analyze serum inflammatory cytokine levels. The findings revealed significant differences in IL-2, tumor necrosis factor-alpha (TNF-α), IL-4, and IL-10 concentrations between the normal pregnancy group and the URSA group before treatment (*P* *<* *0.05*). The URSA group exhibited higher levels of Th1 cytokines and lower levels of Th2 cytokines, signifying an imbalance between Th1 and Th2. Following treatment, the experimental group exhibited significant changes in cytokine levels compared to the control group (*P* *<* *0.05*). Additionally, the treatment success rate was higher in the experimental group at 92.86% (26 out of 28) than in the control group, 72.41% (21 out of 29). These findings revealed the benefits of combining STW with dydrogesterone for improving the immune environment among patients with URSA, correcting Th1/Th2 imbalances, and improving pregnancy outcomes.

#### 4.1.2 Clinical efficacy of pure TCM therapy and internal-external combined therapy with STW

The application of pure TCM, along with both internal and external treatment methods, has illustrated notable clinical effectiveness. He LP et al. (20) reported that the total effective rate of treating threatened abortion of the kidney deficiency type with symptom-modified STW was 94.12% (48, 51), exceeding the rate of the control group treated with only dydrogesterone tablets. Liu WJ (21) conducted an observational study comprising 60 patients with URSA. Compared to the control group (30 patients) treated exclusively with intramuscular progesterone injections, the experimental group (30 patients) received STW combined with syndrome differentiation-based acupoint application, which revealed significant improvements in TCM syndrome scores for patients with URSA during the first trimester of pregnancy. Additionally, the treatment caused an increase in β-HCG, P, and E2 levels.

Furthermore, multiple clinical studies have demonstrated that STW exhibits a favorable safety profile with few adverse reactions in patients (17, 21–23).

#### 4.1.3 Research on quality evaluation of clinical research literature

We performed a structured quality appraisal of the clinical literature, stratified into RCTs and non-randomized controlled trials (NRSIs). These assessments are presented in Supplementary Tables S7, S8. For the detailed risk-of-bias assessment procedure, please refer to the footnotes at the end of Supplementary Table 7.

Quantitative analysis revealed a significant difference in the distribution of bias risk between RCTs and NRSIs (*χ*^2^ test, *P* *<* *0.001*). RCTs predominantly exhibited a “moderate” risk of bias, whereas NRSIs were largely classified as “critical” or “serious”, aligning with the inherent limitations of observational designs regarding confounding control. A common deficiency across both study types was the lack of transparency in reporting, specifically concerning selective outcome reporting and the absence of details regarding randomization procedures (for RCTs) or confounding adjustment (for NRSIs). This suggests that the field has yet to fully adhere to the CONSORT and STROBE reporting guidelines. Regarding study design composition, RCTs constituted the majority of the evidence base (*73.3%, 99/135*), with NRSIs accounting for the remainder (*26.7%, 36/135*). Within the NRSI subgroup, uncontrolled case series represented over 60% of the studies (*21/135* of the total corpus). While the overall landscape is dominated by RCTs, the non-randomized subset is characterized by a significant internal quality disparity: low-level evidence designs (case series and quasi-RCTs) comprise over 80% of the observational studies, highlighting a distinct “quality gap” within the auxiliary evidence base.

### 4.2 Mechanism exploration

Main keywords have been employed to identify significant research fields associated with how STW prevents and treats SA. STW is recognized for its impact on immunological factors, inhibiting cellular apoptosis, supporting embryo viability, and improving the microcirculation in the MFI. By employing antioxidation, cellular repair, immunological modulation, and vascular protection mechanisms, STW significantly mitigates the risk of SA and provides therapeutic benefits.

#### 4.2.1 Rebuilding immune tolerance and building an “immune barrier”

An imbalanced immunological microenvironment significantly contributes to SA in patients (24, 25). In normal pregnancy, Th2-type cellular immunity predominates, characterized by the release of anti-inflammatory cytokines, including IL-10, which promote immune tolerance. Conversely, patients with SA frequently demonstrate hyperactivity of Th1-type immune cells, which secrete pro-inflammatory cytokines such as TNF-α and IFN-*γ*, leading to immunological rejection. The Th1/Th2 imbalance can be rectified by STW through an increase in Th2 cells and a decrease in Th1 cells in peripheral blood (19, 26, 27). This treatment strategy alters the immunological milieu of the endometrium, encourages maternal-fetal immune tolerance, and reduces maternal immune rejection of the semi-allogeneic embryo (28). Mechanistically, directing the Th1/Th2 immune response toward a Th2-dominant phenotype that aligns with pregnancy is crucial for maintaining a seamless pregnancy by enhancing maternal-fetal immune equilibrium. STW influences inflammatory mediators through a bidirectional regulatory mechanism. It downregulates serum pro-inflammatory cytokines, including TNF-α, IL-6, IL-2, and IL-17, and simultaneously upregulates anti-inflammatory cytokines such as IL-10. This adjustment mitigates the harmful effects of inflammatory factors on the integrity of the decidua and the function of trophoblast cells, thereby boosting maternal-fetal immune tolerance, enhancing endometrial receptivity, and aiding in the maintenance of a normal pregnancy (29). In current clinical settings, managing URSA poses a significant challenge, with its pathogenesis being a critical factor in research in gynecology. A previous study (30) demonstrated that STW can enhance the upregulation of anti-inflammatory elements such as IL-10 and antiviral proteins, including tetratricopeptide repeats 4, radical S-adenosyl methionine domain-containing 2, and bone marrow stromal cell Antigen 2. Additionally, it diminishes pro-inflammatory factors such as TNF-α and IFN-α/*γ*, modulating the immune response and stabilizing decidual cells to address URSA (Table 2).

**Table 2:** Mechanistic study of STW on maternal-fetal immune barrier in SA.

Researcher	Medication	Animal model (English Polished)	Inflammatory cytokines & mechanisms
Zhang J et al. (24)	Cuscuta chinensis, Taxillus chinensis, Dipsacus asperoides, and Colla Corii Asini at a weight ratio of 2:1:1:1.	A rat model of ovulation induction with disrupted endometrial immune microenvironment was established: during the late estrus phase, 25 U of Pregnant mare serum gonadotropin (PMSG) was intraperitoneally injected into female rats; 48 h later, 200 U of HCG was administered via the same route to induce superovulation.	IL-4, IL-6, IL-10, LIF, and LIF receptor (LIFR)↑; pro-inflammatory cytokines interferon (IFN)-*γ* and IL-2↓.
Lai M et al. (25)	An herbal extract was prepared using 400 g of Cuscuta chinensis, 200 g of Dipsacus asperoides, 200 g of Taxillus chinensis, and 200 g of Colla Corii Asini.	(1) The SA model established by mating female CBA/J mice with male DBA/2 mice; and (2) a normal pregnancy control model established by mating female CBA/J mice with male BALB/c mice.	Th1-type pro-inflammatory cytokines (IFN-γ, TNF-α)↓; Th2-type anti-inflammatory cytokines (IL-4, IL-10)↑.
Liu Z et al. (27)	Decoction-free herbal granules were prepared following the ratio of Cuscuta chinensis: Taxillus chinensis: Dipsacus asperoides: Colla Corii Asini = 2:1:1:1.	A murine model of controlled ovarian hyperstimulation (COH) characterized by compromised endometrial receptivity was established by employing a long-acting gonadotropin-releasing hormone agonist (GnRHa) pituitary downregulation protocol, with stepwise sequential administration of GnRHa, PMSG, and HCG to trigger robust superovulation.	IL-10, IL-4, and interferon-inducible protein 10 (IP-10)↑; IL-2, IL-17, TNF-α, and IFN-γ↓.
Fang F et al. (28)	Cuscuta chinensis, Dipsacus asperoides, Taxillus chinensis, and Colla Corii Asini—at a weight ratio of 2:1:1:1.	Twenty adult female Sprague-Dawley (SD) rats were used.	TNF-α, IL-6, IL-1β↓; IL-10, along with interferon-stimulated genes (ISGs)↑.

#### 4.2.2 Inhibition of cell death and improvement of embryonic “living conditions”

Oxidative stress primarily entails an excessive generation of reactive oxygen species or an imbalance in the antioxidant defense system (31). It is essential to recognize that it serves as a critical upstream initiator of several cell death mechanisms (32, 33). This activation initiates multiple pathways, including apoptosis, pyroptosis, and ferroptosis, leading to pregnancy loss. Apoptosis primarily affects trophoblast cells and endometrial decidual cells, which are essential for embryo implantation, placental development, and maintenance of pregnancy. Excessive apoptosis in trophoblast cells directly results in embryo implantation failure and developmental arrest. In SA, pyroptosis is a significant link between inflammatory damage and immune system dysfunction. When overactivated, it disrupts pregnancy maintenance mechanisms, affecting immunity, cellular function, and the tissue microenvironment. Ferroptosis, an iron-dependent mechanism different from apoptosis, plays a significant role in placental dysfunction and SA pathogenesis (34) (Table 3).

**Table 3:** Mechanisms of cell death inhibition & embryonic niche optimization.

Researcher	Tx	Model	Pathway	Mechanisms
Lin MK et al. (33)	Cuscuta chinensis & C. campestris extracts, core raw materials of the monarch herb of STW.	Eight-week-old male ICR mice with a body weight ranging from 20 g to 25 g were administered SCOP via intraperitoneal (i.p.) injection at a dose of 1 mg/kg.	SOD and CAT activities and GSH level↑; MDA content↓; IL-1β and TNF-α levels↓; Inhibited excessive acetylcholinesterase (AChE) activation to maintain homeostasis of the central cholinergic system.	Inhibition of oxidative stress-induced neuronal apoptosis.
Park SB et al. (34)	Extract prepared from 20 g of branches (B) of Taxillus yadoriki (TY) parasitic on Neolitsea sericea (NS, hereinafter referred to as TY-NS-B), the core raw material of the ministerial herb in the classic TCM tocolytic formula STW.	Mouse macrophage cell line, RAW264.7 was purchased from Korean Cell Line Bank (Seoul, Korea) and grown in DMEM/F-12 supplemented ith 10% fatal bovine serum (FBS), 100 U/mL penicillin and 100 µg/mL streptomycin.	NF-κB inflammatory pathway & MAPK pathway: TY-NS-B anti-inflammatory effect: ↓ NF-κB activation, ↓ p38/JNK phosphorylation in MAPK pathway, ↓ pro-inflammatory cytokines/inflammatory mediators.	Inhibition of lipopolysaccharide (LPS)-induced pyroptosis and apoptosis in macrophages.
Chen XY et al. (35)	seed, 100 g bark, 100 g Astragalus mong holicus, root, mistletoe, Chinese teasel root, macrocephala, lovage rhizome, and Codonopsis pilosula.	Mice in the TCM treatment group, aspirin treatment group, and control group were injected intraperitoneally with Alarelin, once a day, at 9:00 am in the third day of the estrous cycle. After 9 days, 0.4 mL pregnant mare serum gonadotropin was added and 0.4 mL hCG was injected again 48 h after injection.	B3 and LIF expression↑.	Inhibition of apoptosis in endometrial epithelial cells (EECs).
Qiao ZH et al. (36)	6 g Cuscutae Semen, 3 g Dipsaci Radix et Rhizoma, 3 g Taxilli Herba, and 3 g Asini Corii Colla.	A cellular injury model was established by inducing cytotoxic damage in HTR-8/SVneo human extravillous trophoblast cells with LPS.	NLR family pyrin domain containing 3 (NLRP3)/Caspase-1/Gasdermin D (GSDMD) canonical pyroptosis signaling pathway. NLRP3 may be the key target of STW in inhibiting pyroptosis in the LPS-induced HTR-8/SVneo cell injury model.	Mitigation of LPS-triggered cytotoxic injury in HTR-8/SVneo trophoblast cells.
Lai Y et al. (37)	Cuscuta chinensis Lam, Taxillus sutchuenensis var. duclouxii (Lecomte) H.S.Kiu and Dipsacus L was prepared in the ratio of 4:3:3.	CBA/J mated DBA/2 mice were used to conduct RPL model, while CBA/J mated BALB/c mice were seen as the control group. Mice were orally administered with jianwei Shoutai Pill (JSP), Fer-1 (a ferroptosis inhibitor) or distilled water from day 0.5–12.5 of gestation (GD 0.5–12.5).	Nrf2/HO-1/GPX4 signaling axis governing ferroptosis：GPX4 and SLC7A11↑, which suppress ferroptosis; ACSL4↓, which boosts ferroptosis, and HTR-8/SVneo cell death↓.	Attenuation of ferroptosis in human trophoblast cells.

Increasing evidence indicates that STW supports the normal biological functions of trophoblast and decidual cells. Endowed with antioxidative, anti-inflammatory, and anti-apoptotic activities (35), STW aids in maintaining placental development and ensuring the stability of the maternal-fetal interface (36). Furthermore, STW demonstrates a bidirectional regulatory influence on apoptotic target proteases (11, 12, 37). It enhances the expression of essential anti-apoptotic proteins such as B-cell lymphoma 2 (Bcl-2) and survivin and simultaneously reduces the levels of pro-apoptotic factors, including Bax, Fas/FasL, and caspase-3. This modulation effectively inhibits the apoptotic cascade, hence preventing excessive apoptosis in trophoblast cells. An excess of pyroptosis causes substantial immune cell death and initiates a series of inflammatory responses. The caspase-1 pathway, facilitated by the inflammasome, is the primary mechanism for pyroptosis, with the NLRP3 inflammasome serving as a crucial upstream signal. STW can inhibit NLRP3 activity, decrease caspase-1 cleavage, and interfere with the pyroptotic signaling pathway (12). Consequently, it alleviates inflammatory reactions at the maternal-fetal interface, preventing the propagation of inflammation and further tissue damage. Research conducted by Luo SP and Gao J, employing an RSA mouse model and a trophoblast ferroptosis model, demonstrated that STW may significantly alleviate RSA, potentially by inhibiting ferroptosis. This discovery presents a new method for RSA treatment and elucidates the use of STW in RSA intervention (38).

#### 4.2.3 Optimizing maternal-fetal interface microcirculation and improving blood perfusion

Endometrial receptivity is the endometrium's ability to facilitate embryo implantation, essential for successful implantation and pregnancy establishment (39). In patients with SA, endometrial receptivity is often compromised by factors such as “barren soil” or “microenvironmental imbalance.” Through a multi-target synergistic regulatory mechanism, STW enhances maternal-fetal interface microcirculation, clears “nutrient pathways,” and addresses the “barren soil” issue in patients with SA (37). A previous study (22) has demonstrated that STW increases endometrial thickness, optimizes uterine spiral artery hemodynamics, and facilitates vascular remodeling by upregulating the proportion of type A endometrium and leukemia inhibitory factor (LIF) expression and downregulating the pulsatility index (PI) and resistance index (RI) of uterine spiral arteries. This suggests that STW can collectively restore the “fertile soil” of the endometrium, thereby promoting effective embryo implantation (Table 4).

**Table 4:** STW-mediated regulation of maternal-fetal interface microcirculation and uteroplacental blood perfusion.

Researcher	Year	Intervention	Model	Pathway
Zeng L et al. (40)	2020	The mass ratio of Dipsaci Radix, Taxilli Herba, Cuscutae Semen to Asini Corii Colla is 2:1:1:1.	The normal pregnancy model was established by mating CBA/J female mice with BALB/C male mice, and the RSA model was established by mating CBA/J female mice with DBA/2 male mice.	VEGF/VEGFR2 angiogenesis pathway, coagulation-fibrinolysis balance regulatory pathway.
Huang X et al. (9)	2024	Cuscutae Semen, Taxilli Herba and Dipsaci Radix were crushed into coarse powder and mixed at a mass ratio of 2:1:1.	Clinical villous tissues were collected from 20 women with unplanned normal pregnancy and 20 patients with spontaneous abortion at the Day Surgery Center of the First Affiliated Hospital of Guangzhou University of Chinese Medicine from March 2022 to March 2023 (Ethical Approval No.: K-2022-046).	PI3K/AKT/mTOR signaling pathway.
LI J X et al. (42)	2024	The prescription consisted of Cuscutae Semen 30 g, Dipsaci Radix 15 g, Taxilli Herba 15 g, processed Asini Corii Colla 15 g, Salviae Miltiorrhizae Radix et Rhizoma 15 g, and Angelicae Sinensis Radix 15 g.	A classic animal model of recurrent spontaneous abortion (RSA) was established by mating inbred CBA/J female mice with inbred DBA/2 male mice.	Wnt/β-catenin signaling pathway.

Subsequently, vascular endothelium dysfunction may cause placental hypoxia and ischemia (40), thereby increasing the risk of SA. STW safeguards vascular endothelial cells by increasing the concentrations of molecules such as heat shock protein 27 (HSP27) and *α*-enolase, thereby mitigating endothelial damage and preserving vascular balance (41). Furthermore, vascular endothelial growth factor (VEGF) is essential in regulating vascular function at the maternal-fetal interface (42). Insufficient VEGF expression levels hinder trophoblast invasion into the vascular wall, disrupting normal vascular remodeling and compromising the stability of embryo implantation. Therefore, clinical studies have demonstrated that the protein levels of VEGFA, VEGFR1, VEGFR2, and key kinases in the PI3K/AKT/mTOR signaling pathway (PI3K, phosphorylated AKT [p-AKT], and phosphorylated mTOR [p-mTOR]) are markedly diminished in villous tissues from patients with SA compared to those from women with normal, uncomplicated pregnancies (*P* *<* *0.05, P* *<* *0.01*). *In vitro* investigations using a mifepristone-induced HTR-8/SVneo cell injury model demonstrated a significant reduction in the protein expression of angiogenesis-related molecules, specifically VEGFA, VEGFR1, and VEGFR2 (*P* *<* *0.01, P* *<* *0.05*), alongside a notable decrease in PI3K pathway-related protein expression (*P* *<* *0.01*). Notably, treatment with modified STW significantly reversed the downregulation of these angiogenesis-related proteins and the key mediators of the PI3K/AKT/mTOR signaling pathway within this cell model (*P* *<* *0.01, P* *<* *0.05*) (9). Besides, STW has been observed to downregulate both mRNA and protein expression of hypoxia-inducible factor 1*α* (HIF-1*α*) and simultaneously enhance VEGF expression and its receptors. This regulatory mechanism promotes decidual angiogenesis and improves local blood perfusion at the maternal-fetal interface, which constitutes a fundamental mechanism supporting and sustaining successful pregnancy (43).

### 4.3 Research on target tissue, cells, and molecules

MFI is a complex framework that integrates essential elements from the maternal and fetal perspectives. The placental trophoblast layer and villous mesenchyme specifically originate from the fetal side, whereas the maternal side contains decidual tissue, immune cells, and vascular endothelial cells (44, 45). Emerging evidence indicates that STW exerts therapeutic effects primarily at the MFI (46, 47) (Table 5).

**Table 5:** Regulatory signaling pathways of STW.

Researcher	Year	Intervention	Model	Pathway	Study type
Mu ZN et al. (13)	2023	12 g Cuscutae Semen (Tu Si Zi), 6 g Taxilli Herba (Sang Ji Sheng), 6 g Dipsaci Radix (Xu Duan), and 6 g Asini Corii Colla (E Jiao).	Forty female CBA/J mice were mated with male mice at a 2:1 ratio: fifteen male DBA/2 mice were used to establish a recurrent spontaneous abortion (RSA) mouse model (30 mice total), while five male BALB/c mice were used to establish a normal pregnancy model (10 mice total).	JAK1/STAT3 signaling pathway.	Basic Experimental Study.
Zhou CL et al. (51)	2025	/	“Drug-compound-target” network.	EGFR/PI3K/AKT signaling pathway.	Network Pharmacology and Experimental Validation Study.
Wu HW et al. (51)	2018	Total flavones were extracted from the dried seeds of Cuscuta chinensis Lam. (the monarch herb of classic STW formula).	Female Sprague-Dawley (SD) rats (body weight: 269.55 ± 22.35 g) and male SD rats (body weight: 428.15 ± 22.55 g) were used as experimental animals.	MAPK signaling pathway (ERK/p38/JNK sub-pathways).	Basic Experimental Study.

In keyword co-occurrence analysis, “decidua” and “trophoblast cells” are identified as prominent terms owing to their high frequency, as illustrated in Figure 5 and Supplementary Table S4. This section examines research specifically concentrated on these two critical components, whereas related subjects are addressed in the section on mechanisms. Trophoblast cells play an essential role in maintaining pregnancy by facilitating embryonic invasion, hormone secretion, and material exchange. Abnormalities in their proliferation, invasion, hormone secretion, immune regulation, and vascular remodeling are significant pathological factors in SA, especially RSA. Guided by the principle of “tonifying the kidney and replenishing essence,” the STW approach seeks to restore trophoblast cell function and rectify their abnormal state through six targeted strategies: (1) mitigating excessive apoptosis to maintain cellular integrity (11, 12, 35, 37, 38); (2) enhancing proliferation and invasion capabilities and promoting β-HCG secretion to facilitate implantation (18, 21); (3) optimizing energy metabolism to support hormone production (18, 21); (4) fostering immune tolerance to prevent immune responses (19, 26, 27); (5) promoting vascular remodeling to enhance blood flow (9, 43); (6) repairing signaling pathways to stabilize cell function (9, 13, 48–50).

The decidua, a specialized tissue derived from the endometrium and affected by pregnancy hormones, is essential for embryo implantation. It regulates immunological tolerance, nutrient exchange, hormonal responses, and signal transduction, offering foundational support for pregnancy. Incomplete decidualization, abnormal cellular activity, and disrupted immune and metabolic balance are significant pathological factors in SA. STW facilitates embryo implantation and sustains pregnancy by restoring decidual structure and functionality and optimizing the microenvironment (15, 51). Additionally, research demonstrates that STW can modulate protein expression in mouse decidual tissue (52), helping maintain pregnancy and reduce embryo loss rates.

Molecular target research has focused on elucidating the impact of signaling pathway dysregulation, a core pathogenic mechanism of SA, on the development of a complex signal transduction network. Aberrant inhibition or activation of pathways, including PKB, MAPK, FOXO3-PR, STAT3, and NF-κB, may lead to trophoblast dysfunction, incomplete decidualization, immune imbalance, and vascular complications, contributing to pregnancy loss. Integrating network pharmacology predictions with experimental or proteomic validation enables a comprehensive verification of key protein targets (48). STW restores signal transduction equilibrium by synergistically influencing multiple pathways, thereby clarifying its conventional role in “tonifying the kidney and preventing miscarriage” (9, 13, 49, 50).

## 5 Discussion

Based on the above measurement findings, this study identifies three major challenges in the research of Shoutai Pills: ① Insufficient evidence-based registration in clinical research. ② Fragmented mechanism research. ③ The integration of traditional Chinese medicine theory and microscopic mechanisms still needs further exploration. The specific issues and future research directions are as follows:

### 5.1 Limitations of the clinical evidence

As outlined in Section 3.2, elaborated in detail in Section 4.1, and corroborated by the risk-of-bias assessments of included studies presented in Supplementary Tables S7, S8, current clinical research on STW is predominantly restricted to regional research clusters in China, with a significant deficiency of multicenter, large-sample, and meticulously standardized RCTs. Major international guidelines assert that the current high-quality evidence is insufficient to validate the efficacy and long-term safety of TCM therapies, including compound herbal prescriptions, for the treatment of spontaneous abortion. Consequently, TCM is not recommended as a first-line or routine clinical intervention. Despite this, complementary and alternative medicine is widely utilized globally for managing pregnancy loss. There is an urgent need for rigorously designed multicenter, large-sample RCTs to confirm the therapeutic efficacy and safety profile of these interventions. Clinicians in practice should fully inform patients opting for TCM about the limitations of existing evidence, systematically monitor hepatic and renal function during treatment, and mitigate risks associated with concurrent use of Western pharmaceuticals. Future cross-regional collaborative research is essential to generate robust evidence, address gaps in international guidelines, and advance the global translation and standardization of evidence-based TCM tocolytic therapies.

### 5.2 Fragmented mechanistic research

Section 4.2 delineates the recent advancements in mechanistic research, yet specific unresolved issues persist, hindering further investigation of related mechanisms. Mechanistic research on STW is advancing; however, it predominantly adheres to a reductionist “single-pathway, single-target” model. It primarily focuses on local biological factors such as inflammatory cytokines, immunological regulation, trophoblast apoptosis, and oxidative stress. This fragmented approach fails to clarify STW's broad-spectrum effects in the complex maternal-fetal interface ecosystem. As a multi-component TCM formula, STW has “multi-target, multi-level, and multi-process” pharmacological traits. Its biological essence pertains more to the system-level homeostasis modulation than to linear interaction with individual receptors. To comprehend STW's complex mechanisms, research methods such as multi-omics integration and network pharmacology are essential. Multi-omics technologies, including genomics, transcriptomics, proteomics, and metabolomics, provide an extensive and comprehensive view of biological systems (53, 54), and network pharmacology elucidates the TCM formula's multi-target and multi-pathway actions.

### 5.3 Absence of a syndrome-endotype mapping model

The therapeutic efficacy of TCM in reproductive medicine has garnered international recognition (55, 56). In TCM, RSA is predominantly attributed to kidney deficiency; hence, enhancing kidney function is prioritized in therapeutic strategies to stabilize pregnancy. In TCM, the concept of the kidney extends beyond the anatomical organ recognized in Western medicine, encompassing a vital energy system that regulates the innate origin of life, physical development, and reproductive function and is responsible for storing the essence of life, referred to as kidney essence. The idea stated in the Huangdi Neijing, which posits that the kidney regulates reproduction, indicates that reproductive capacity, conception, and fetal sustenance are intrinsically linked to the kidney (57). The TCM concept of the “kidney-kidney essence-uterus” axis demonstrates functional similarities with the hypothalamic-pituitary-ovarian axis in modern medical science (58). STW the classic anti-abortion formula, exerts efficacy by replenishing kidney essence and consolidating Chong-Ren channels to stabilize pregnancy, which forms the theoretical basis for the syndrome clustering results in this study.

However, co-occurrence clustering analysis in this study highlights a gap between TCM syndromes such as “kidney deficiency” and modern pathophysiological markers, indicating that existing research frameworks struggle to integrate traditional TCM concepts with complex biological processes.

### 5.4 Strategic future directions

(1) Establishing a high-quality, cross-institutional, multicenter, and worldwide clinical evidence system to dismantle regional evidence silos and conduct RCTs and real-world studies with global generalizability. A recent clinical study on Zishen Yutai Pill, a classic TCM gynecological formula derived from STW, was published in Nature Communications (59). This formula was developed by Professor Luo Yuankai, a distinguished national gynecologist and the father of Professor Luo Songping from our research team, and its publication has demonstrated the significant potential of TCM in improving live birth rates. (2) Creating a “syndrome-pathological mechanism-molecular target” mapping model to integrate TCM theory with modern biology, enabling mechanism-driven clinical applications (60, 61). (3) Mapping system-level biological landscapes of the maternal-fetal interface to clarify the cellular, tissue, and molecular networks involved in STW intervention through multi-omics integration and network pharmacology, establishing a robust framework for understanding compound formula pharmacology (62, 63). (4) Improving cross-regional, cross-disciplinary, and international research collaboration by promoting data exchange, biobank development, and standardized research protocols to facilitate the internationalization of the research system (64, 65).

## 6 Limitations

The bibliometric analysis conducted in this study has several inherent structural limitations. First, this bibliometric analysis reflects the publication activity and research attention in this field, not the strength or quality of clinical evidence. The frequency of publication does not equate to the robustness of therapeutic efficacy or mechanistic findings. Meanwhile, the analysis relies on database coverage, potentially introducing structural sampling bias; data that utilize non-standard terminology or are derived from unpublished datasets may be excluded. Second, the literature selected for this study is predominantly concentrated in China, a reflection of the disciplinary focus on traditional Chinese medicine compound prescriptions, which naturally leads to research being primarily conducted within the region. Despite the inclusion of major international databases such as WoS and PubMed, the findings indicate a notable scarcity of international scientific collaboration in this domain. Third, it should be emphasized that, despite this systematic risk-of-bias appraisal of included studies, the present study cannot establish the clinical efficacy of STW, nor can it confirm any proposed putative mechanisms. Bibliometric methods produce a “knowledge map” instead of an “evidence hierarchy.” The identified hotspots and trends should be viewed as structural representations of domain cognition rather than as rankings of treatment efficacy or mechanistic robustness.

## 7 Conclusion

It should be emphasized that the added value of this bibliometric analysis over a conventional narrative review lies in four uniquely bibliometric contributions: (1) algorithmically derived temporal phase demarcation via timeline clustering (Figure 7B), which objectively partitions 44 years of research into three non-overlapping developmental stages; (2) burst detection (Figure 8) that quantitatively pinpoints emerging frontiers—such as “aerobic glycolysis” and “modified shoutai wan”—with measurable burst strengths; (3) network topology analysis (Figures 3–5) that reveals a core–periphery collaboration structure and identifies bridge keywords (e.g., HSP27, IL-17) through betweenness centrality metrics; and (4) a macro-level quality gap (*χ*^2^*, P* *<* *0.001*) revealed by systematic RoB stratification across 135 clinical studies. These structural and temporal patterns are not derivable from qualitative synthesis alone.

Our analysis suggests that STW research is at a critical moment of paradigm restructuring. This field is poised to change significantly from empirical medicine to mechanism-driven medicine by reconstructing clinical evidence systems, incorporating systems biology approaches, modernizing theoretical frameworks, and expanding international collaborative networks. Systematic reviews targeting specific clinical outcomes such as miscarriage, as well as more in-depth basic research, need to be continued in subsequent studies. This study aims to sort out the overall research context in the field of STW.

## Data Availability

The original contributions presented in the study are included in the article/Supplementary Material, further inquiries can be directed to the corresponding author/s.
