# Supplementary material for: Global research status and emerging frontiers of Shoutai Wan in the prevention and treatment of spontaneous abortion: a bibliometric analysis (1982–2026)
Source: Front Med (Lausanne). 2026 Oct 13;13:1850663. doi: 10.3389/fmed.2026.1850663 (PMC13710286; doi:10.3389/fmed.2026.1850663)
Supplement: Supplementary file 1 [file Datasheet1.docx]

**Table S1 Search strategy.**

| **Search** | **Search Formula** | **Results** | **Data** |
| --- | --- | --- | --- |
| 1. CNKI, VIPDatabase, Wanfang Database | SU=(spontaneous abortion+pregnancy loss+recurrent spontaneous abortion, RSA+threatened abortion+biochemical pregnancy+habitual abortion+fetal leakage+fetal restlessness+inevitable abortion+incomplete abortion+complete abortion+missed abortion+spetic abortion) AND SU=Shoutai Wan | 1929 | 2026/1/30 |
| 2. PubMed | (Shoutai Wan[Title/Abstract] OR STW[Title/Abstract] OR ShouTai Pill[Title/Abstract] OR ShouTai Pills[Title/Abstract]) AND (Spontaneous Abortion[MeSH Terms] OR spontaneous abortion[Title/Abstract] OR SA[Title/Abstract] OR miscarriage*[Title/Abstract] OR Recurrent Spontaneous Abortion[MeSH Terms] OR RSA[Title/Abstract]) AND (prevention[Title/Abstract] OR treat*[Title/Abstract]OR therapy[Title/Abstract] OR management[Title/Abstract]) | 34 | 2026/1/30 |
| 3. Web of Science (WOS) | (Shoutai Wan OR STW OR ShouTai Pill OR ShouTai Pills) AND (spontaneous abortion OR SA OR miscarriage* OR recurrent spontaneous abortion OR RSA) AND (prevention OR treat* OR therapy OR management) | 35 | 2026/1/30 |
