# Supplementary material for: Global research status and emerging frontiers of Shoutai Wan in the prevention and treatment of spontaneous abortion: a bibliometric analysis (1982–2026)
Source: Front Med (Lausanne). 2026 Oct 13;13:1850663. doi: 10.3389/fmed.2026.1850663 (PMC13710286; doi:10.3389/fmed.2026.1850663)
Supplement: Supplementary file 2 [file Datasheet2.docx]

**Table S2 Specific information on institution.**

| **ID** | **label** | **weight<Links>** | **weight<Total link strength>** | **score<Avg. citations>** |
| --- | --- | --- | --- | --- |
| 2607 | Hunan University of Chinese Medicine | 21 | 653 | 36.2492 |
| 822 | Guangzhou University of Chinese Medicine | 21 | 320 | 22.6369 |
| 1909 | Hebei University of Chinese Medicine | 16 | 126 | 10.5556 |
| 1799 | Chengdu University of Traditional Chinese Medicine | 18 | 274 | 36.5048 |
| 362 | Shandong First Medical University | 20 | 163 | 26.1818 |
| 287 | Zhejiang Chinese Medical University | 20 | 201 | 33.1212 |
| 1989 | Jiangxi Provincial Maternal and Child Health Hospital | 19 | 193 | 48.2542 |
| 2048 | Journal of Hunan University of Chinese Medicine | 20 | 199 | 52.5517 |
| 2620 | Journal of Traditional Chinese Medicine | 18 | 188 | 42.2632 |
| 418 | Zhejiang University of Technology | 18 | 190 | 53.3036 |
| 688 | Guangzhou Medical University | 20 | 183 | 27.2679 |
| 1448 | Guangdong Medical University | 19 | 151 | 53.9412 |
| 2066 | Haining Second People's Hospital | 19 | 64 | 24.375 |
| 2587 | Hebei Provincial Maternal and Child Health Care Center | 14 | 69 | 24.2727 |
| 2661 | Heilongjiang University of Chinese Medicine | 18 | 92 | 50.6977 |
| 774 | Huizhou Hospital of Traditional Chinese Medicine | 17 | 155 | 37.3571 |
| 3428 | Meizhou Hospital of Traditional Chinese Medicine | 17 | 71 | 5.3659 |
| 78 | Shandong University of Traditional Chinese Medicine | 12 | 43 | 4.4211 |
| 106 | Shenzhen Seventh People's Hospital | 15 | 55 | 4.6471 |
| 1166 | Henan University of Chinese Medicine | 19 | 83 | 29.2424 |
| 1183 | Beijing University of Chinese Medicine | 18 | 105 | 36 |
| 1623 | Liaoning University of Traditional Chinese Medicine | 17 | 80 | 32.4688 |
