# Supplementary material for: Global research status and emerging frontiers of Shoutai Wan in the prevention and treatment of spontaneous abortion: a bibliometric analysis (1982–2026)
Source: Front Med (Lausanne). 2026 Oct 13;13:1850663. doi: 10.3389/fmed.2026.1850663 (PMC13710286; doi:10.3389/fmed.2026.1850663)
Supplement: Supplementary file 3 [file Datasheet3.docx]

**Table S3 Shoutai Wan related research team cooperation unit.**

| **ID** | **Core high frequency unit** | **Cooperation unitCore high frequency unit** |
| --- | --- | --- |
| 1 | Guangzhou University of Chinese Medicine | The first affiliated hospital, the first clinical medical college, lingnan medical research center, nursing college, basic medical college, The First Hospital of Wuhan and Women's and Children's Hospital Affiliated to Qingdao University. |
| 2 | Hebei University of Traditional Chinese Medicine | Key Laboratory of Integrated Traditional Chinese and Western Medicine for Liver and Kidney Diseases, College of Integrated Traditional Chinese and Western Medicine, Graduate School, Collaborative Innovation Center of Integrated Traditional Chinese and Western Medicine for Reproductive Diseases, Affiliated Hospital. |
| 3 | Hunan University of Traditional Chinese Medicine | College of Integrated Traditional Chinese and Western Medicine, Hunan University of Chinese Medicine, The First Affiliated Hospital of Hunan University of Chinese Medicine, Hebei Maternity and Child Health Care Center, Molecular Pathology Laboratory and Hunan University of Chinese Medicine. |
