# Supplementary material for: Global research status and emerging frontiers of Shoutai Wan in the prevention and treatment of spontaneous abortion: a bibliometric analysis (1982–2026)
Source: Front Med (Lausanne). 2026 Oct 13;13:1850663. doi: 10.3389/fmed.2026.1850663 (PMC13710286; doi:10.3389/fmed.2026.1850663)
Supplement: Supplementary file 4 [file Datasheet4.docx]

**Table S4 Top 30 High-frequency keywords.**

| **ID** | **Keywords** | **Frequency** | **Centrality** | **Year** |
| --- | --- | --- | --- | --- |
| 1 | threatened abortion | 48 | 0.47 | 2009 |
| 2 | recurrent abortion | 28 | 0.34 | 2012 |
| 3 | recurrent spontaneous abortion | 17 | 0.17 | 2008 |
| 4 | recurrent miscarriage | 14 | 0.27 | 2011 |
| 5 | shoutai wan | 13 | 0.33 | 2014 |
| 6 | spontaneous abortion | 12 | 0.26 | 2011 |
| 7 | modified shoutaiwan | 12 | 0.52 | 2015 |
| 8 | early threatened abortion | 11 | 0.18 | 2013 |
| 9 | fetal restlessness | 10 | 0.1 | 2013 |
| 10 | fetal leakage | 9 | 0.01 | 2013 |
| 11 | jianwei shoutai wan | 9 | 0.4 | 2016 |
| 12 | habitual abortion | 8 | 0.22 | 2013 |
| 13 | maternal fetal interface | 5 | 0.16 | 2009 |
| 14 | kidney deficiency type | 5 | 0.29 | 2019 |
| 15 | acupoint application | 5 | 0.11 | 2016 |
| 16 | pregnancy outcome | 4 | 0.16 | 2016 |
| 17 | heat shock protein 27 | 4 | 0.11 | 2013 |
| 18 | decidual tissue | 4 | 0.04 | 2014 |
| 19 | shoutaiwan/treatment application | 4 | 0 | 2008 |
| 20 | endometrial receptivity | 4 | 0.13 | 2018 |
| 21 | low molecular weight heparin | 4 | 0.1 | 2021 |
| 22 | aerobic glycolysis | 4 | 0.17 | 2023 |
| 23 | il 17 | 3 | 0.02 | 2012 |
| 24 | blocking antibody | 3 | 0.06 | 2011 |
| 25 | kidney deficiency syndrome | 3 | 0.05 | 2018 |
| 26 | hormone levels | 3 | 0.03 | 2021 |
| 27 | traditional chinese medicine | 3 | 0.05 | 2016 |
| 28 | oxidative stress | 3 | 0.08 | 2020 |
| 29 | therapeutic efficacy | 3 | 0 | 2015 |
| 30 | clinical efficacy | 3 | 0.32 | 2013 |
