# Supplementary material for: Global research status and emerging frontiers of Shoutai Wan in the prevention and treatment of spontaneous abortion: a bibliometric analysis (1982–2026)
Source: Front Med (Lausanne). 2026 Oct 13;13:1850663. doi: 10.3389/fmed.2026.1850663 (PMC13710286; doi:10.3389/fmed.2026.1850663)
Supplement: Supplementary file 5 [file Datasheet5.docx]

**Table S5 Specific information on keyword clustering.**

| **ID** | **Label** | **Silhouette** | **Average Clustering Year** | **Relevant terms** |
| --- | --- | --- | --- | --- |
| #0 | recurrent abortion | 0.993 | 2019 | recurrent abortion (16.86, 1.0E-4); lactic acid (8.29, 0.005); decidual tissue (8.29, 0.005); threatened abortion (4.78, 0.05); network pharmacology (4.74, 0.05) |
| #1 | spontaneous abortion | 0.895 | 2020 | spontaneous abortion (24.93, 1.0E-4); regulatory t cells (9.78, 0.005); helper t cells 17 (9.78, 0.005); jianwei shoutaiwan (9.78, 0.005); jianwei shoutai wan (6.14, 0.05) |
| #2 | shoutai wan | 0.966 | 2021 | shoutai wan (14.82, 0.001); habitual abortion (14.77, 0.001); aerobic glycolysis (6.14, 0.05); acidic microenvironment (4.86, 0.05); clinical efficacy (4.86, 0.05) |
| #3 | traditional chinese medicine | 0.994 | 2016 | traditional chinese medicine (13.58, 0.001); pregnancy outcome (13.58, 0.001); frozen thawed embryo transfer (6.71, 0.01); efficacy observation (6.71, 0.01); early stage (6.71, 0.01) |
| #4 | maternal fetal interface | 0.985 | 2017 | maternal fetal interface (12.69, 0.001); morinda officinalis (6.28, 0.05); diethyl phthalate (6.28, 0.05); hypoxia inducible factor 1 (6.28, 0.05); rats (6.28, 0.05) |
| #5 | acupoint application | 0.948 | 2016 | acupoint application (12.19, 0.001); estradiol (e2) (6.04, 0.05); human chorionic gonadotropin ( hcg) (6.04, 0.05); combined with immunotherapy (6.04, 0.05); recurrent miscarriage of unknown etiology (6.04, 0.05) |
| #6 | decidua | 1 | 2018 | decidua (12.97, 0.001); membranoprotein a2 (6.41, 0.05); thyroxine binding protein (6.41, 0.05); western blot (6.41, 0.05); enolase (6.41, 0.05) |
| #7 | hormone levels | 0.987 | 2013 | hormone levels (7.26, 0.01); advanced maternal age (7.26, 0.01); low molecular weight heparin (7.26, 0.01); combined therapy (7.26, 0.01); chinese herbal fumigation and washing (7.26, 0.01) |
| #8 | oxidative stress | 0.963 | 2022 | oxidative stress (8.76, 0.005); immune factors (8.76, 0.005); di (2 ethylhexyl) phthalate (8.76, 0.005); embryonic development (8.76, 0.005); shoutai wan (2.89, 0.1) |
