# Supplementary material for: Global research status and emerging frontiers of Shoutai Wan in the prevention and treatment of spontaneous abortion: a bibliometric analysis (1982–2026)
Source: Front Med (Lausanne). 2026 Oct 13;13:1850663. doi: 10.3389/fmed.2026.1850663 (PMC13710286; doi:10.3389/fmed.2026.1850663)
Supplement: Supplementary file 6 [file Datasheet6.docx]

**Table S6 Specific Medication in Clinical Studies.**

| **Researcher** | **Composition** | **The Control Group** | | **The Experimental Group(Conventional Medicine)** |
| --- | --- | --- | --- | --- |
|  |  | **Decoction Method** | **Dosage and Administration** |  |
| Shi HY et al. [17] | 10 g Taxilli Herba, 20 g Cuscutae Semen, 10 g Dipsaci Radix, 10 g Asini Corii Colla. | Decoction-free granules, provided by Sichuan Xinlüsè Pharmaceutical Technology Development Co., Ltd. | One dose was administered daily, mixed with 300 mL of water, and divided into three equal portions (100 mL each) for consumption in the morning, noon, and evening, with continuous administration for 6 consecutive weeks. | One dose was administered daily, mixed with 300 mL of water, and divided into three equal portions (100 mL each) for consumption in the morning, noon, and evening, with continuous administration for 6 consecutive weeks. |
| Yao X et al. [18] | 10 g Asini Corii Colla, 10 g Taxilli Herba, 10 g Dipsaci Radix, 15 g Cuscutae Semen. | Five hundred milliliters (500 mL) of water was added, and the herbs were soaked for 30 minutes prior to decoction. | Decoct until the solution reaches 300 mL for oral administration, with a 2-week treatment course. | Decoct until the solution reaches 300 mL for oral administration, with a 2-week treatment course. |
| Shi HY et al.[19] | 10 g Taxilli Herba, 20 g Cuscutae Semen, 10 g Dipsaci Radix, and 10 g Asini Corii Colla in the form of decoction-free granules. | Decoction-free granules. | The regimen was 1 dose per day mixed with 300 mL of water, taken in three 100-mL servings (morning, noon, and evening) for a 6-week consecutive course. | The regimen was 1 dose per day mixed with 300 mL of water, taken in three 100-mL servings (morning, noon, and evening) for a 6-week consecutive course. |
| He LP et al. [20] | 25 g Taxilli Herba, 20 g each of Cuscutae Semen, Dipsaci Radix, and Asini Corii Colla. | Add water and proceed with decoction of the herbs. | Decoct the herbs until the solution reduces to 400 mL, and take it orally twice a day (once in the morning and once in the evening). | Decoct the herbs until the solution reduces to 400 mL, and take it orally twice a day (once in the morning and once in the evening). |
| Liu WJ [21] | 15 g Taxilli Herba, 15 g Codonopsis Radix, 12 g Cuscutae Semen, 10 g Dipsaci Radix, 10 g Asini Corii Colla [melted and taken with boiling water], 10 g Atractylodis Macrocephalae Rhizoma. | The herbs were prepared by water decoction for oral intake. | The regimen was 1 dose per day, split into morning and evening doses, with treatment terminated once the pregnancy reached the 12th week. | The regimen was 1 dose per day, split into morning and evening doses, with treatment terminated once the pregnancy reached the 12th week. |
|  | Acupoint application, the specific acupoints selected were Guanyuan (Conception Vessel 4, CV4) and bilateral Shenshu (Bladder Meridian 23, BL23). | Ten grams each of Cuscuta chinensis, Taxillus chinensis, Dipsacus asperoides, Eucommia ulmoides, Codonopsis pilosula, Astragalus membranaceus, and Boehmeria nivea root were ground into powder, blended with water to make an ointment, and then applied. | Application: once daily, 6–8 h/session; remove within 8 h if obvious skin flushing; discontinued after 12th gestational week. | Application: once daily, 6–8 h/session; remove within 8 h if obvious skin flushing; discontinued after 12th gestational week. |
